# Association of Selective Serotonin Reuptake Inhibitor Use With Abnormal Physical Movement Patterns as Detected Using a Piezoelectric Accelerometer and Deep Learning in a Nationally Representative Sample of Noninstitutionalized Persons in the US

**DOI:** 10.1001/jamanetworkopen.2022.5403

**Published:** 2022-04-07

**Authors:** Michael V. Heinz, George D. Price, Franklin Ruan, Robert J. Klein, Matthew Nemesure, Aliza Lopez, Nicholas C. Jacobson

**Affiliations:** 1 Center for Technology and Behavioral Health, Dartmouth College, Lebanon, New Hampshire; 2 Department of Psychiatry, Dartmouth-Hitchcock Medical Center, Lebanon, New Hampshire

## Abstract

**Importance:**

Selective serotonin reuptake inhibitors (SSRIs) are a common first-line treatment for some psychiatric disorders, including depression and anxiety; although they are generally well tolerated, SSRIs have known adverse effects, including movement problems, sleep disruption, and gastrointestinal problems (eg, nausea and upset stomach). No large-scale studies using naturalistic, longitudinal, objective data have validated physical activity findings, and actigraphy data are well suited to address this task.

**Objectives:**

To evaluate whether differences in physical movement exist among individuals treated with SSRIs compared with control participants and to identify the unique features of the movement of patients treated with SSRIs.

**Design, Setting, and Participants:**

This cross-sectional study examines longitudinally collected wearable movement data within a cross-sectional sample of 7162 participants from the 2005-2006 National Health and Nutrition Examination Survey (NHANES), a nationally representative population-based sample of noninstitutionalized persons in the US having both medication information and passive movement data. Statistical analysis was performed from April 1, 2021, to February 1, 2022.

**Exposures:**

The use of SSRIs (sertraline hydrochloride, escitalopram oxalate, fluoxetine hydrochloride, paroxetine hydrochloride, and citalopram hydrobromide), as reported by participants interviewed by NNHANES personnel, was the primary exposure, measured as a binary variable (taking an SSRI vs not taking an SSRI).

**Main Outcomes and Measures:**

The primary outcome was the intensity of body movement as recorded by a piezoelectric accelerometer worn on the right hip for more than 1 week.

**Results:**

Of the 7162 participants included in the study, the mean (SD) age was 33.7 (22.6) years, 266 (3.7%) were taking an SSRI, 3706 (51.7%) were female, 1934 (27.0%) were Black, 1823 (25.5%) were Mexican American, 210 (2.9%) were other Hispanic, 336 (4.7%) were other or multiracial, and 2859 (39.9%) were White (per the NHANES data collection protocol). A cross-validated, deep learning classifier was constructed that achieved fair performance predicting SSRI use (area under the curve, 0.67 [95% CI, 0.64-0.71] for the validation set and 0.66 [95% CI, 0.64-0.68] for the test set). To account for possible confounding by indication, we constructed a parallel model incorporating depression severity, finding only marginal performance improvement. When averaged across individuals and across 7 days, the results show less overall movement in the SSRI group (mean, 120.1 vertical acceleration counts/min [95% CI, 115.7-124.6 vertical acceleration counts/min]) compared with the non-SSRI control group (mean, 168.8 vertical acceleration counts/min [95% CI, 162.8-174.9 vertical acceleration counts/min]).

**Conclusions and Relevance:**

This cross-sectional study found a moderate association between passive movement and SSRI use, as well as SSRI detection capacity of passive movement using time series deep learning models. The results support the use of passive sensors for exploration and characterization of psychotropic medication adverse effects.

## Key Points

**Question:**

What can digital technologies and deep learning indicate about the association between the use of antidepressants and abnormal physical movement patterns?

**Findings:**

This cross-sectional study found a significant association between antidepressant use and measured movement, even when accounting for depression. Individuals treated with antidepressants had an overall lower level of physical activity.

**Meaning:**

This study highlights the utility of data collected for the exploration and characterization of the adverse effects of medication and suggests the importance of future prospective research aimed at further understanding the association between antidepressant use and movement.

## Teaser

This cross-sectional study evaluates whether differences in physical movement exist between individuals taking selective serotonin reuptake inhibitors (SSRIs) and control participants and identifies the unique features of the movements of patients treated with SSRIs.

## Introduction

Selective serotonin reuptake inhibitors (SSRIs) have become the first-line pharmacotherapy for managing some of the most common psychiatric conditions, including depression and anxiety.^1,2^ Given the prevalence of SSRI use, the potential adverse effects of SSRIs are of considerable importance for public health. Although SSRIs have a much improved adverse effect profile compared with their predecessors, the monoamine oxidase inhibitors and the tricyclic antidepressants, clinical evidence from the past several decades suggests that SSRIs are not benign, with self-reported data suggesting that 38% of patients experienced adverse effects and 25% of these patients reported that their adverse effects were a significant burden.^3^ Common adverse effects of SSRIs have included impaired sexual functioning (56%), drowsiness (53%), weight gain (49%), dry mouth (19%), insomnia (16%), fatigue (14%), nausea (14%), and dizziness (13%). In addition, 12% of patients have reported adverse effects associated with involuntary physical movement or “tremor.” Numerous case reports have also highlighted the potential SSRI-mediated adverse effects associated with abnormal physical movement (often termed *extrapyramidal symptoms*), including dystonia, akathisia, and tremor.^4,5,6,7^

We see 2 problems in the existing literature on the adverse effects of SSRIs that have clinical implications. First, existing investigations may provide an incomplete picture of the adverse effect profile of SSRIs, given that research to date is based almost solely on either case studies or studies commissioned by drug manufacturers. This incomplete understanding of its adverse effect profile poses clinically significant challenges in that adherence to antidepressant use is affected by both patient and clinician factors. For instance, adherence to the use of such medications is reduced by insufficient patient education about antidepressants,^8^ which may include adverse effects. Drug manufacturers may not be sufficiently motivated to explore the more subtle (although still bothersome) adverse effects that are not likely to be life threatening. Both existing case studies and industry-sponsored studies have relied almost exclusively on retrospective self-reported symptoms. This is a problem because, for an adverse effect to be detected, patients must be consciously aware of the adverse effect, and in case studies, patients or physicians must also attribute the adverse effect to the drug. In such contexts, subtler adverse effects may go undetected.

Second, there are empirical and theoretical reasons to suspect that SSRIs may affect not only finer, localized physical movement, such as tremor or other forms of extrapyramidal symptoms, but also more generalized physical movement. For instance, multiple experiments have shown an association between SSRI administration and a generalized reduction in the capacity for bodily movement.^9,10,11^ Research of this kind is consistent with the central fatigue hypothesis,^12^ which suggests that increased serotonin in the brain can lead to an earlier onset of fatigue under exertion. Improved knowledge of such movement-related adverse effects associated with the use of SSRIs would be beneficial because this drug class is not generally associated with reduced physical movement, and such knowledge could better inform health care professionals about the potentially problematic downstream effects of these drugs. Such knowledge may also prevent these movement-related problems associated with SSRIs from being misdiagnosed, unobserved, or untreated.

We propose that new and more rigorous research is needed to investigate the movement-related adverse effects associated with the use of SSRIs. In this domain, longitudinal data obtained from an ambulatory device (ie, a piezoelectric accelerometer) are ideal. Such data have the potential to capture more objective, time-dependent, naturalistic, nonconscious features that manifest within daily life. Movement data of this type have been used in multiple studies examining responses to psychiatric medications, including antipsychotics,^13^ stimulants,^14,15^ and antidepressants,^16,17^ although, to our knowledge, no large-scale, naturalistic studies have examined the association of SSRI use with movement. Longitudinal activity data may provide information about an individual’s diurnal patterns, daily physical activity, and sleep disturbance. In addition to general physical activity, there is also reason to believe that previously the reported adverse effects of SSRI use, including involuntary movements, drowsiness, insomnia, fatigue, and weight gain, would be detectable in the longitudinal data.

With the existing National Health and Human Nutrition Examination Survey (NHANES) data set, the present analysis aims to better understand the association between SSRI use and physical movement by using dense longitudinal activity data and machine learning methods in a data-driven approach. Our primary aims are (1) to evaluate whether SSRI use is associated with abnormal physical movement patterns in day-to-day life and (2) to characterize the nature of any temporal movement patterns found to be associated with SSRI use. We hypothesized that, given the reported movement-related adverse effects of SSRIs (eg, sleep changes, drowsiness, and tremors), the longitudinal data on movement would predict SSRI use with moderate accuracy, even while considering confounders of indication (ie, depression).

## Methods

### Study Design

The present analysis uses a cross-sectional design, with a sample from the 2005-2006 NHANES. The study was approved by the National Center of Health Statistics research ethics review board, and written informed consent was obtained from participants prior to data collection. This study followed the Strengthening the Reporting of Observational Studies in Epidemiology (STROBE) reporting guideline.

### Participants

**Figure 1.**
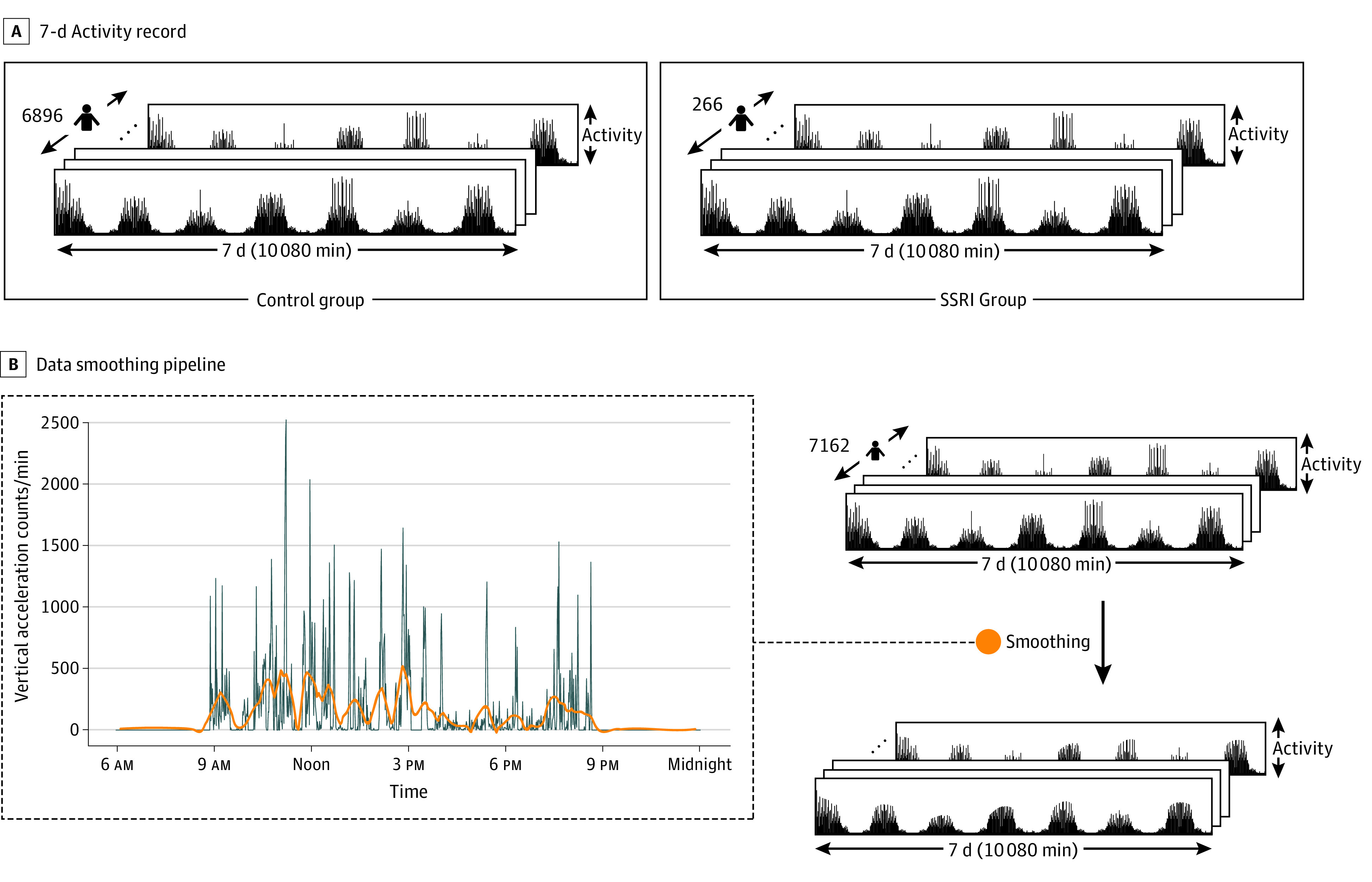
Data Set and Smoothing A, Schematic of the 7-day activity record for each participant across the control and selective serotonin reuptake inhibitor (SSRI) groups. The control group contains 6896 individuals, and the SSRI group contains 266 individuals. B, Schematic of the data smoothing pipeline. The left side of the diagram shows a sample of the smoothed activity superimposed on the movement data for 24 hours for 1 participant. After smoothing, there is less noise in the data, allowing for better visualization of trends. The blue line indicates no filter, and the orange line indicates smoothing.

The 2005-2006 NHANES was used to survey a total of 10 348 randomly selected participants in a nationally representative sample. Of these participants, a subset of 7162 participants (3456 male participants [48.3%] and 3706 female participants [51.7%]; mean [SD] age, 33.7 [22.6] years) had reliable 7-day passive movement intensity data and prescription medication information. The NHANES is a major program of the National Center of Health Statistics aimed at assessing the health and nutrition status of the US population, through survey and objective data. The NHANES uses US Census data to sample noninstitutionalized individuals residing in the United States.^18^ Race and ethnicity information was self-reported by participants and collected as part of a routine demographic survey. Movement intensity data were collected via a hip-mounted ActiGraph AM-7164 piezoelectric accelerometer (ActiGraph LLC).^19^ The acceleration data were summed within 1-minute epochs spanning 7 days (for a total of 10 080 movement data points for each participant). Medications were self-reported by participants and, in 55.1% of cases (9921 of 18 005), verified by prescription bottle.^20^ In total, 266 persons reported taking SSRI medications, including sertraline hydrochloride, escitalopram oxalate, fluoxetine hydrochloride, paroxetine hydrochloride, and citalopram hydrobromide. Actigraphy data (longitudinal time series) collected over 1 week constitute the study’s dependent (outcome) variable, and data on SSRI use (binary: yes or no) constitute the study’s independent (exposure) variable. Potential confounders included demographic features and indication (eg, depression severity, measured via self-report). Our data set is shown schematically in Figure 1A.

### Data Preprocessing

#### Data Smoothing

To reduce noise and outliers in the longitudinal data, we applied the Savitzky-Golay filter, a polynomial smoothing filter (Figure 1B)^21^ found to better preserve temporal data, with a signal to noise ratio 2 times higher than a simple moving mean filter.^22^

#### Reshaping Data and Standardization

We reshaped participant movement data according to the method described by Rahman and Adjeroh^23^ and used in processing NHANES movement data. For each participant, we reshaped the 1-dimensional (10 080 minutes) array to a sequence of 7 daily actigraphy arrays (of size 24 hours × 60 minutes) (Figure 1A). As discussed by Rahman and Adjeroh,^23^ this representation explicates potential temporal patterns in the data across hours and days. Finally, we standardized each minute of the activity data across all 7162 participants using *z*-score normalization.

### Data Splitting and Stratification

We used a 10-fold cross-validation approach (80%), with a single held-out test set (20%). Because of a drug class imbalance (SSRI vs no SSRI), we used stratification during data splitting to maintain similar proportions of each class in both the training and held-out test sets. Similarly, during cross-validation, we used StratifiedKFold^24^ from Python’s sklearn package to maintain similar class proportions. To mitigate the potential learning bias from class imbalance, we applied class weights (266 of 7162 for the no-SSRI class and 6896 of 7162 for the SSRI class) to enforce a greater model penalty for poor predictions in the SSRI class.

### Model Pipeline and Analysis

#### Simple Logistic Regression With Wavelet-Derived Features

To begin modeling, we started with a simple approach using logistic regression with wavelet-derived features to act as a baseline for accuracy and model stability. Using Daubechies wavelet,^25^ we performed 6 levels of decomposition, extracting the mean value, percentiles (25th and 75th), entropy, SD, variance, and the mean number of crossings. We used these features in a 10-fold cross-validated logistic regression model.

#### Convolutional–Long Short-Term Memory Model With Time Series Data

**Figure 2.**
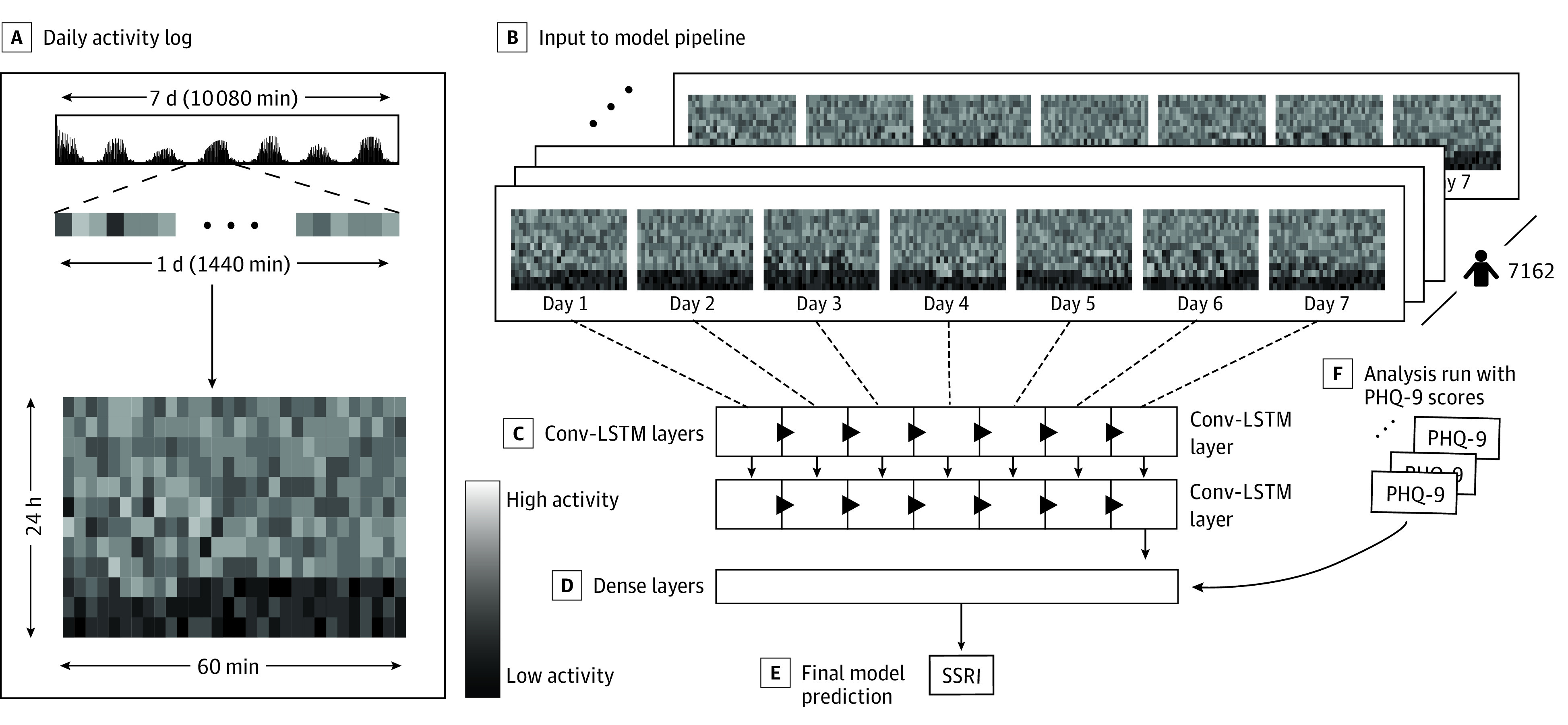
Convolutional–Long Short-Term Memory (Conv-LSTM) Pipeline A, The reshaping of the data can be visualized by representing each minute as a box, with the shading inversely associated with the magnitude of activity at that point. A daily activity log is constructed by creating a 60 × 24 matrix, where each row represents 60 minutes. B, The input to the model pipeline comprised a (7162 × 60 × 24 × 7) activity array. This was passed through 2 Conv-LSTM layers (C) and then to a dense layer (D), which outputs the final model prediction (E). F, Analysis was run with 9-item Patient Health Questionnaire (PHQ-9) scores by concatenating the PHQ-9 score to output from the Conv-LSTM layer. SSRI indicates selective serotonin reuptake inhibitor.

After testing a simple logistic regression model, we constructed a deep learning model, capable of encoding time series data to compare performance. We began by reshaping our data to a sequence of 7 daily actigraphy arrays for each participant (Figure 2A and B). Our deep learning model comprised multiple convolutional–long short-term memory (Conv-LSTM) layers (Figure 2C)^26^ and dense layers (Figure 2D). Long short-term memory networks and convolutional neural networks have shown promise in modeling time series data.^23,27,28,29,30^ To exploit the favorable properties of both LSTMs and convolutional neural networks for time series, we used Conv-LSTM layers, conceptualized in 2015 by Shi et al^26^ and successfully used by Rahman and Adjeroh.^23^ This approach allows for a reduction in the number of LSTM time steps from 10 080 (ie, 1 time step for every minute) to 7 (ie, 1 time step for every day of the week).

**Figure 3.**
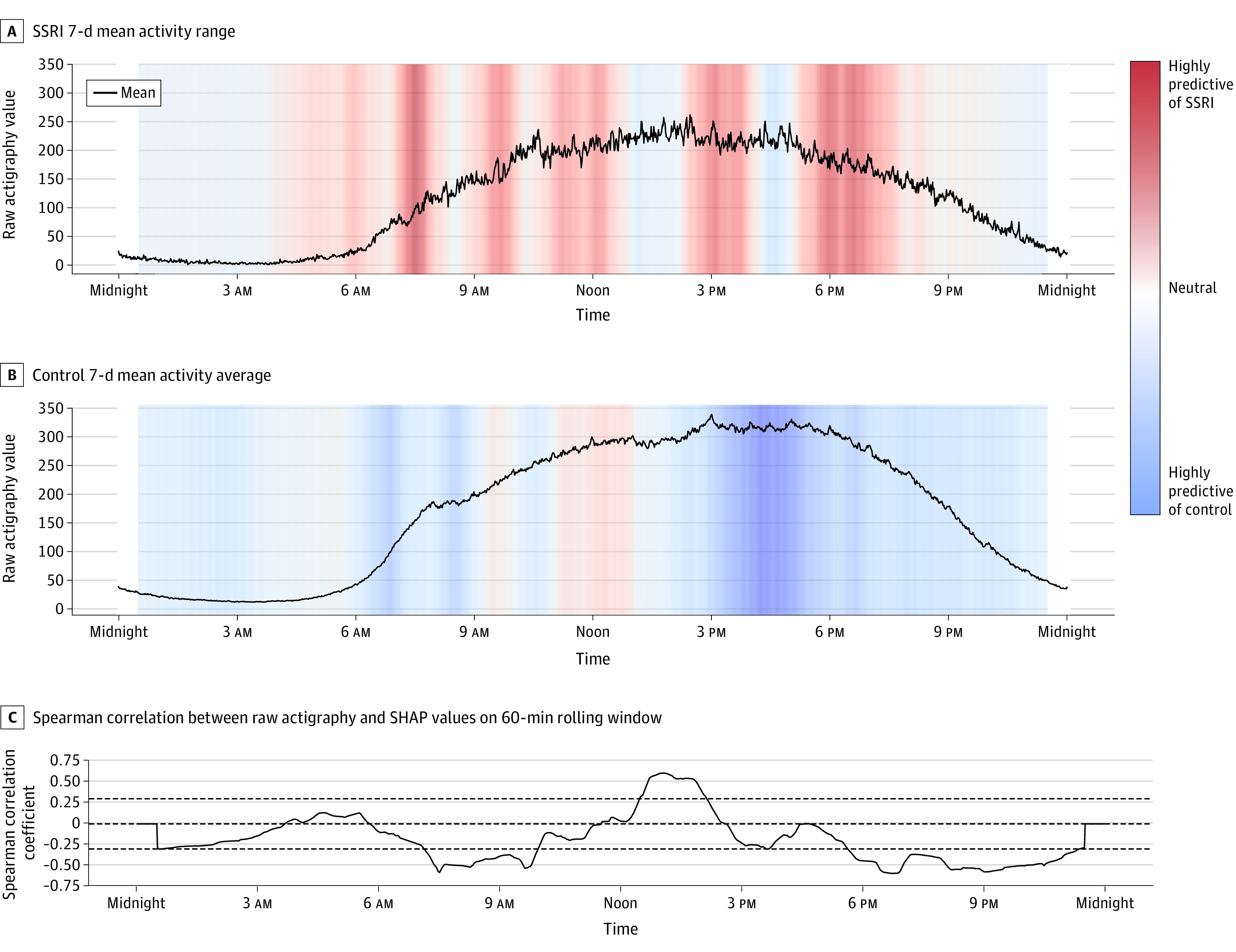
Shapley Additive Explanations (SHAP) Values for Actigraphy Modeling A, Activity magnitude for participants in the selective serotonin reuptake inhibitor (SSRI) group over time. B, Activity for control participants. The curves (A and B) indicate the mean movement across the respective groups across weeks. These curves were plotted against colored SHAP values, where red regions correspond to high SHAP values and blue regions correspond to low SHAP values. High or red SHAP values suggest a positive association between movement and SSRI use, and low or blue SHAP values suggest a negative association between movement and SSRI use. Regions of relatively high or low SHAP values reveal time frames that were influential to the model’s prediction. C, Spearman correlation between participants’ raw actigraphy values and corresponding SHAP values for every minute in a day, smoothed with a 60-minute rolling window mean value.

We began by passing the data to a Conv-LSTM layer (Figure 2C), a maximum pooling layer, a second Conv-LSTM layer, and a dropout layer (dropout rate, 0.2). The output was then passed to a dense layer (Figure 2D) (similar to the approach used by Rahman and Adjeroh^23^) to make a final scalar prediction: the likelihood of being on an SSRI ∈ [0, 1] (Figure 2E). Shapley Additive Explanations (SHAP) were averaged across the 10 folds of the deep learning model and used to quantify the relative importance of a given time point (minute) across participants and across days in our model.^31^ To visualize the relative association of the actigraphy data with the prediction of SSRI use, SHAP values were averaged over a 60-minute rolling window and plotted on a background color scale against SSRI and control activity averaged across days and participants of their respective group (Figure 3A and B).

### Statistical Analysis

The χ^2^ test was performed for sex and for race and ethnicity, and the *t* test was performed for the mean 9-item Patient Health Questionnaire (PHQ-9) total score and mean age. The χ^2^ test was 1-sided, the *t* test was 2-sided, and results were deemed statistically significant at *P* < .05. Statistical analysis was performed from April 1, 2021, to February 1, 2022, using the SciPy package in Python, version 1.4.1.

## Results

Baseline demographic and clinical information for participants is shown in Table 1. Of the 7162 participants included in the study, the mean (SD) age was 33.7 (22.6) years, 266 (3.7%) were taking an SSRI, 3456 (48.3%) were male, and 3706 (51.7%) were female. A total of 1934 participants (27.0%) were Black, 1823 (25.5%) were Mexican American, 210 (2.9%) were other Hispanic, 336 (4.7%) were other or multiracial, and 2859 (39.9%) were White (per the NHANES data collection protocol).

**Table 1.** Baseline Demographic Characteristics of Participants in the 2005-2006 National Health and Nutrition Examination Survey SSRI Cohort and the Non-SSRI Cohort^a^

Characteristic	**Participants, No. (%)**		*P* value
	SSRI (n = 266)	**No SSRI (n = 6896)**	
Age, y			
<20	29 (10.9)	2943 (42.7)	<.001
20-29	24 (9.0)	851 (12.3)	
30-39	25 (9.4)	696 (10.1)	
40-49	43 (16.2)	685 (9.9)	
50-59	56 (21.1)	513 (7.4)	
60-69	40 (15.0)	561 (8.1)	
70-84	45 (16.9)	542 (7.9)	
≥85	4 (1.5)	105 (1.5)	
Mean (SD) age, y	49.6 (20.2)	33.1 (22.4)	
Sex			
Female	186 (69.9)	3520 (51.0)	<.001
Male	80 (30.1)	3376 (49.0)	
Race and ethnicity			
Non-Hispanic			
Black	34 (12.8)	1900 (27.6)	<.001
White	180 (67.7)	2679 (38.8)	
Mexican American	33 (12.4)	1790 (26.0)	
Other Hispanic	7 (2.6)	203 (2.9)	
Other race (including multiractial)^b^	12 (4.5)	324 (4.7)	
Clinical			
Mean (SD) PHQ-9 total score	4.26 (4.27)	2.68 (2.82)	<.001

Abbreviations: PHQ-9, 9-item Patient Health Questionnaire; SSRI, selective serotonin reuptake inhibitor.

^a^ The χ^2^ test was performed for sex and race and ethnicity, and the *t* test was performed for mean PHQ-9 total score and mean age.

^b^ This category indicates racial categories not matching 1 of the predefined categories or multiracial.

### Model Performance Metrics

We report our results using multiple metrics for (1) the wavelet-derived feature logistic regression model, (2) the Conv-LSTM model fit to actigraphy data alone, and (3) the Conv-LSTM model fit to actigraphy data concatenated with depression scores in Table 2. We found fair model performance overall, with marginal area under the receiver operating characteristic curve (AUC) gain in the Conv-LSTM model compared with the logistic regression model (Conv-LSTM model: mean AUC, 0.67 [95% CI, 0.64-0.71] for the validation set and 0.66 [95% CI, 0.64-0.68] for the test set; logistic regression model: mean AUC, 0.65 [95% CI, 0.60-0.71] for the validation set and 0.64 [95% CI, 0.64-0.64] for the test set). We found higher variability in the cross-validated logistic regression model compared with the Conv-LSTM model, indicating lower model stability. Across all models, we found moderate sensitivity and specificity (Table 2), high negative predictive value, very low positive predictive value, and a high population stability index (lower in the logistic regression model compared with the Conv-LSTM model).

**Table 2.** Modeling Performance Metrics

Model^a^	AUC, mean (95% CI)	Cut point	Sensitivity	Specificity	PPV, %	NPV, %	BAC	PSI
Conv-LSTM model								
Held-out test set (activity only)	0.66 (0.64-0.68)	0.48	0.79	0.50	5.77	98.43	0.65	10.02
Cross-validation sets (activity only)	0.67 (0.64-0.71)	0.49	0.68	0.60	6.90	98.10	0.64	10.03
Conv-LSTM model with depression scores								
Held-out test set (PHQ-9 plus activity)	0.66 (0.65-0.67)	0.50	0.74	0.56	6.13	98.26	0.65	10.10
Cross-validation sets (PHQ-9 plus activity)	0.70 (0.65-0.75)	0.50	0.73	0.60	7.10	98.35	0.66	10.20
Wavelet LogReg model								
Held-out test set	0.64 (0.64-0.64)	0.08	0.72	0.56	5.95	98.15	0.64	3.40
Cross-validation sets	0.65 (0.60-0.71)	0.06	0.72	0.54	6.05	98.22	0.63	3.41

Abbreviations: AUC, area under the receiver operating characteristic curve; BAC, balanced accuracy; Conv-LSTM, convolutional–long short-term memory; LogReg, logistic regression; NPV, negative predictive value; PHQ-9, 9-item Patient Health Questionnaire; PPV, positive predictive value; PSI, population stability index.

^a^ Given the small size of our selective serotonin reuptake inhibitor group, we present these metrics for each model (Conv-LSTM run with movement data alone, Conv-LSTM run with movement data and depression scores, and logistic regression operating on wavelet-derived features) to ensure comprehensive report of model performance. Sensitivity, specificity, PPV, NPV, BAC, and PSI are mean values across 10 cross-validation sets or across distinct model runs of held-out test set; AUC remains the primary outcome metric, which is discussed in the Results and Discussion sections.

### Movement Differences Between SSRI and Control Groups

When averaged across individuals and across 7 days (Figure 3A and B), our results show overall less movement in the SSRI group (mean, 120.1 vertical acceleration counts/min [95% CI, 115.7-124.6 vertical acceleration counts/min]) compared with the non-SSRI control group (mean, 168.8 vertical acceleration counts/min [95% CI, 162.8-174.9 vertical acceleration counts/min]). We also found important differences in the rate of movement change in the morning and evening hours, with the SSRI group showing a slower morning increase in movement and a slower evening decrease in movement. This difference can be observed in Figure 3A and B and was quantified by calculating the slopes of the best-fit lines over morning and evening intervals (6-9 am and 6-9 pm, respectively). The SSRI group showed a morning slope of 0.73 vertical acceleration counts/min^2^ (95% CI, 0.72-0.74 vertical acceleration counts/min^2^) compared with 0.97 vertical acceleration counts/min^2^ (95% CI, 0.95-0.99 vertical acceleration counts/min^2^) in the control group. Comparably, the SSRI group showed an evening slope of −0.39 vertical acceleration counts/min^2^ (95% CI, −0.38 to −0.40 vertical acceleration counts/min^2^) compared with −0.76 vertical acceleration counts/min^2^ (95% CI, −0.75 to −0.77 vertical acceleration counts/min^2^) in the control group.

### Addressing the Potential for Confounding by Indication

Given the prescription of SSRIs primarily for mood and anxiety disorders, we encountered the potential for confounding by indication. To address this potential confounder, we used PHQ-9 depression severity scores,^32^ available for 59.8% (857 of 1433) of our held-out test participants. We imputed missing PHQ-9 scores with participant demographic characteristics using multivariate imputation.^33^ Using our deep learning pipeline, we included information on depression as input to the first dense layer in our model (Figure 2), by concatenating PHQ-9 scores with activity data. We found no model improvement in the test set AUC and a very marginal increase in the validation set AUC. This finding suggested little additive value of PHQ-9 scores in incrementing the prediction of SSRI use. In addition, we have included results from a general logistic regression model (eTable in the Supplement), directly comparing standardized β coefficients between depression severity and movement. The results demonstrate higher weight of movement compared with depression severity in predicting SSRI use, further supporting that the SSRI predictions were not confounded by indication.

### Model Explainability

To address the question of model explainability, we used SHAP to assess the relative impotance of features, in which each time point was considered a feature, and each participant’s movement intensity at the respective time point was considered an instance. Results are shown in Figure 3A and B.

Features (ie, time points) with high relative importance for detecting SSRI use are shown in different shades of red, while those with high importance in not detecting SSRI use (control) are shown in different shades of blue (Figure 3A and B). We found that the activity points in the morning and early afternoon are particularly important in positively detecting SSRI use (Figure 3B) and that activity in the afternoon and evening is important in negatively detecting SSRI use (Figure 3A and B). In addition, we present the Spearman correlation between the SHAP value and movement intensity at each minute (Figure 3C) to show the association between raw movement intensity and the respective SHAP value (ie, the importance of the feature).

## Discussion

To date, understanding of the adverse effects of SSRIs is based largely on patient self-report, often via retrospective surveys^3^ and reports from the pharmaceutical industry. Although we identified a single, small study examining the association between SSRI use and movement,^17^ to our knowledge, no large-scale studies to date have examined the association between SSRI use and movement in an ecologically valid, naturalistic way. The present study uses objective, dense longitudinal data, passively collected over 7 days from a large nationally representative sample, and examines the associations between SSRI use and physical movement profiles. Our results demonstrate associations between SSRI use and human movement and further demonstrate the existence of a movement phenotype characteristic of SSRI use.

Among individuals taking SSRIs, we found (1) overall less movement across 7 days, (2) more gradual increases in movement in the morning, and (3) a more gradual decrease in movement intensity in the evening (Figure 3A and B). The overall decreased intensity of movement in the SSRI group is consistent with the central fatigue hypothesis,^12^ which emphasizes the importance of monoamine neurotransmitters, such as serotonin and dopamine, in regulating physical activity and fatigue. Robust evidence from animal studies suggests decreased performance (ie, shorter time to fatigue) in response to increased serotonergic activity.^34^ Although similar results have been found in humans,^35^ the outcomes are more mixed and the evidence less robust.^12^ This association between SSRI use and overall decreased movement is especially important considering the benefits associated with physical activity and exercise for individuals with mild to moderate depression.^36^ Supposing a causal link between SSRI use and movement (although experimental prospective research is needed to establish this), the positive effect of SSRI use on depression may be attenuated by lower physical activity levels for some patients.

Our results also suggest that evening and overnight actigraphy patterns are associated with non-SSRI model inference (Figure 3B). This association may suggest a link between nighttime-specific motor changes and SSRI use and may be contextualized in the literature suggesting SSRI-induced sleep architecture disruption^37^ as well as reports of SSRI-worsened insomnia.^3^ Taken together, the present findings begin to disentangle the complex, potentially confounding association between movement, medication, and depression. It suggests that SSRIs are associated with unique movement phenotypes, independent of one of their main indications.

### Limitations

There are several important limitations that should be considered in this research. First, our results demonstrate detection only, not causation. However, such naturalistic detection studies are important in prompting future randomized prospective research on this subject. Second, our outcome variable is subject to confounding by indication. We mitigated as much as possible by incorporating participant depression scores into our results. Because depression scores were available for only 59.8% of the sample, we used a robust multivariate approach to impute missing values. Severity scores for anxiety, obsessive-compulsive disorder, and posttraumatic stress disorder (and other potential SSRI indications) were not assessed in the study, and it is possible that some of the abnormal movement patterns are due to these indications rather than the medication alone. Third, our data set does not contain information about SSRI adherence or dosage. We are, therefore, not able to make dose- or adherence-associated inferences. Fourth, the SSRI group was small compared with the control group; however, we accounted for this by more highly weighting those in the SSRI cohort. Our results indicate minimal AUC loss for model performance on the held-out test set. Fifth, individuals were not randomly assigned to the SSRI or control group; therefore, subgroup baseline differences may exist (age, mean PHQ-9 score, sex, and race and ethnicity are all possible confounding variables).

## Conclusions

To our knowledge, our research is the first to investigate adverse effect profiles of SSRIs using naturalistic, passively collected, longitudinal data. Our results demonstrate an overall reduction in movement among those taking SSRIs compared with those who are not taking SSRIs. In addition, individuals prescribed SSRIs seem to have overall slower increase in movement in the morning and likewise a slower tapering of movement in the evening, suggesting less well-defined sleep-wake boundaries. Our results highlight the utility of passively collected data for the exploration and characterization of the adverse effects of medications and suggest the importance of future prospective research aimed at further understanding the association between antidepressants and movement.

## Notes

Supplement. **eTable.** Standardized Beta Coefficient Estimates for Both Movement and Depression Score in a General Logistic Regression Model
