# Supplementary material for: Association of Selective Serotonin Reuptake Inhibitor Use With Abnormal Physical Movement Patterns as Detected Using a Piezoelectric Accelerometer and Deep Learning in a Nationally Representative Sample of Noninstitutionalized Persons in the US
Source: JAMA Netw Open. 2022 Apr 7;5(4):e225403. doi: 10.1001/jamanetworkopen.2022.5403 (PMC8990330; doi:10.1001/jamanetworkopen.2022.5403)
Supplement: Supplement. — eTable. Standardized Beta Coefficient Estimates for Both Movement and Depression Score in a General Logistic Regression Model [file jamanetwopen-e225403-s001.pdf]

Supplementary Online Content

Heinz MV, Price GD, Ruan F, et al. Association of selective serotonin reuptake inhibitor use with abnormal physical movement patterns as detected using a piezoelectric accelerometer and deep learning in a nationally representative sample of noninstitutionalized persons in the US. *JAMA Netw Open.* 2022;5(4):e225403. doi:10.1001/jamanetworkopen.2022.5403

eTable. Standardized Beta Coefficient Estimates for Both Movement and Depression Score in a General Logistic Regression Model

This supplementary material has been provided by the authors to give readers additional information about their work.

© 2022 Heinz MV et al. *JAMA Network Open.*

eTable. Standardized Beta Coefficient Estimates for Both Movement and Depression Score in a General Logistic Regression Model. Standard error and P value for each estimate are also shown. Both are statistically significant.

	Beta	*Standard Error*	*P-Value*
Movement	0.72	0.22	0.0010*
Depression Score	0.40	0.11	0.0002*

© 2022 Heinz MV et al. *JAMA Network Open.*
